# Factors associated with self-reported suboptimal antiretroviral adherence and limited retention in care among people living with HIV who attend a large ART clinic in Panama City, Panama

**DOI:** 10.1371/journal.pone.0311048

**Published:** 2024-11-27

**Authors:** Amanda Gabster, Félix Díaz Fernández, Juan Miguel Pascale, Angelique Orillac, Samuel Moreno-Wynter, Casey D. Xavier Hall, Eugenia Flores Millender, Frank (´Frankie´) Wong, Mónica Jhangimal, Anyi Yu-Pon, Cristel Rodríguez-Vargas, Diógenes Arjona-Miranda, Bárbara Fuentes, Germán Henostroza, Ana Belén Araúz

**Affiliations:** 1 Instituto Conmemorativo Gorgas de Estudios de la Salud, Panama; 2 National Research System, National Secretariat of Science, Technology and Innovation, Panama City, Panama; 3 Center of Population Sciences for Health Equity, College of Nursing, Florida State University, Tallahassee, FL, United States of America; 4 Instituto de Investigaciones Científicas y Servicios de Alta Tecnología AIP, Panama City, Panama; 5 Hospital Santo Tomás, Panama City, Panama; 6 Universidad de Panamá, Facultad de Medicina, Campus Universitario, Panama City, Panama; 7 College of Social Work, Florida State University, Tallahassee, FL, United States of America; 8 Florida State University–Republic of Panama campus, Panama City, Panama; 9 Universidad Interamericana de Panama, Facultad de Medicina, Panama City, Panama; 10 Division of Infectious Diseases, University of Alabama Birmingham, Birmingham, AL, United States of America; University of the Witwatersrand Johannesburg Faculty of Health Sciences, SOUTH AFRICA

## Abstract

**Background:**

The prevalence of HIV in Panama is estimated to be 1.0%; only 71% of individuals on antiretroviral treatment (ART) were virally suppressed in 2022. This study aimed to describe the prevalence of suboptimal adherence (≥1 missed doses in previous four weeks) and limited retention in HIV care (≥1 missed HIV care appointments in previous 12 months) among adults (aged ≥18 years) who attended the most populous urban ART Clinic in Panama City.

**Methods:**

In this cross-sectional study, participants completed a self-administered questionnaire. Univariable and bivariable analyses were used to describe the prevalence of suboptimal adherence and limited retention in HIV care. Multivariable logistic regression identified factors associated with suboptimal adherence at p<0.05.

**Results:**

We included 375 participants (209 identified as men, 158 as women, 8 another gender). Of those who responded, 37.3% (n = 125/335) reported suboptimal adherence: 28.6% (n = 53/185) of men, 49.0% (n = 71/145) women, 20.0% (n = 1/5) another gender; p<0.01; 18.6% (n = 69/371) reported limited retention in care:13.6% (n = 28/206) men, 24.2% (n = 38/157) women, 37.5% (n = 3/8) another gender, p = 0.01. In multivariable analyses, suboptimal adherence was associated with gender (49.0%women vs. 28.6% men, AOR = 1.86, 95%CI:0.97–3.57), depressive symptoms:46.2% severe symptoms vs. 28.1% minimal-mild, AOR = 2.19,95%CI:0.96–5.04), and lifetime intimate partner emotional violence (IPV) 48.2% vs.no emotional IPV 32.2%, OR = 1.96,95%CI:1.15–2.90, and lifetime physical IPV 46.9% vs.no physical IPV 32.6%, OR = 1.82, 95%CI:1.15–2.90. In unadjusted analyses, limited retention in care was associated with gender (24.2%women vs.13.6% men OR = 2.03, 95%CI:1.18–3.49), difficulty paying rent/mortgage/utilities (22.6% vs.14.9% no difficulty paying, OR = 1.67,95%CI = 0.98–2.83); no variables were associated in the multivariable model.

**Conclusions:**

This study found high prevalence of suboptimal ART adherence and limited retention in care, especially among women; these factors were associated with severe depressive symptoms, as well as lifetime emotional and physical IPV. These results show the need for integrated mental health and IPV intervention for all individuals, including focused support for women.

## Introduction

Human Immunodeficiency Virus (HIV) infection and care is a global public health challenge. Globally, an estimated 39 million people were living with HIV (PLHIV) in 2022 [1]. Antiretroviral therapy (ART) has transformed HIV care; HIV can now be managed as a chronic disease if the viral load is controlled. However, suboptimal adherence to ART, defined as missing one or more doses in the past month, and limited retention in care, defined as missing one or more scheduled HIV care appointments in the past year, have been associated with morbidity and premature mortality [2–4]. In 2020, UNAIDS released the ambitious 95-95-95 targets to be met by 2030. These goals emphasize that 95% of people living with HIV are aware of their status, 95% of those diagnosed to receive sustained ART, and 95% of those on treatment to achieve viral suppression by 2030. The last 95 highly depends on individuals taking their medication as directed and attending their clinical appointments, including viral load testing [5, 6]. The 2020 90-90-90 goals were met with uneven global success [7]. For example, treatment coverage was limited for men (compared to women) and children compared to adults [7, 8]. A summary of the commitments and targets within the UN Assembly’s 2021 Political Declaration on HIV and AIDS acknowledges that inequalities are hindering reaching the goals. Furthermore, the 2021 Declaration calls to reduce to no more than 10% the number of women and girls who experience gender-based inequalities and sexual and gender-based violence by 2025 [9].

Adherence to ART medication and retention in HIV care are two factors that are interrelated but are independently associated with HIV health-related outcomes, including viral suppression, reduced ART resistance, survival, and decreased ongoing transmission [10–12]. Suboptimal ART adherence can lead to increased viral replication, the development of drug resistance, and an overall decline in health [13, 14]. Limited retention in HIV care can further exacerbate these challenges by missing viral load testing and control of comorbidities, including the monitoring of secondary effects of ART psychological and psychiatric disorders. There are several reasons for suboptimal adherence and poor retention in care. Discrimination at the individual and social levels may directly hinder attendance to care appointments and adherence to ART [15, 16]. Additionally, some regimens may have secondary effects, which may deter individuals from continuing medication [17].

Panama’s HIV prevalence was 1.0% in 2022 [18]; nationwide, opportunistic infections related to advanced HIV remain the ninth cause of mortality [19]. Few data describe ART adherence and retention in Panama. Most recently, when analyzing UNAIDS’ cascade goals in 2018, Panama reached 70%-76%-76%, while adjusting for all PLHIV, only 54% were on treatment, and only 41% were virally suppressed [20]. In 2022, the country reached 83%-79%-71%, whilst adjusting for all PLHIV in Panama, only 66% were on treatment, and 47% were virally suppressed [21]. In 2022, at the most populous urban ART clinic in the country, where the present study took place, of all individuals who had been diagnosed and were to attend this clinic, 58% of these were in treatment, and 75% of those in treatment were virally suppressed (Unpublished data, Ministry of Health, 2023). Despite the limited progress, the country has structurally made strides to combat the HIV epidemic. Since 2016, ART and HIV care have been free to everyone in the country ([22], Law 40 of August 14, 2018).

Panama has recently transitioned to a combination ART regimen of tenofovir, lamivudine, and dolutegravir (TLD) (Unpublished data, Ministry of Health, 2022). Since the transition in 2020, 90% of individuals at the largest ART clinic in Panama City are currently using TLD. This regimen has been endorsed as the preferred first-line treatment by the World Health Organization (WHO) [23], due to dolutegravir’s historically high barrier to ART resistance [24] and good tolerability [25], compared to other regimens. Worryingly, in 2024, WHO reported an increase in dolutegravir resistance worldwide, possibly due to adherence difficulties [26]. Despite the advantages of TLD rollout, compared to efavirenz-based treatment, virological failure is still greater in patients who use TLD and maintain <90% ART adherence (3 or more missed doses in 4 weeks), compared to those who maintain >95% adherence (1 or fewer missed doses) [25, 27]. Furthermore, among individuals, even with low viral load, non-perfect adherence to combination therapy such as TLD has been associated with increased cardiovascular events, inflammation, immune activation, and mortality [28–30].

Although data exist on the percentage of individuals in the largest ART clinic in Panama who have access to ART and are virally suppressed, there are currently no studies that have been undertaken that describe the sociodemographic factors related to suboptimal ART adherence and limited retention in HIV care in this clinic. This analysis aimed to understand some demographic, partnership, and psychosocial factors related to suboptimal adherence to ART and limited retention in HIV care. Understanding these factors is imperative for designing interventions that aim to increase adherence and retention among individuals living with HIV in Panama City.

## Methods

We conducted a cross-sectional study among adults (aged ≥18 years) living with HIV who attended a large urban ART clinic. Men and women were included at different periods in 2022 due to funding limitations for the primary outcome of describing the sexually transmitted infection prevalence among participants. The current analysis only focuses on self-reported ART adherence and limited retention in care variables. The study included individuals assigned male at birth (AMaB [cisgender men, transgender women, and intersex individuals assigned male at birth]) between February 22 and March 25, 2022, and among individuals assigned female at birth (AFaB [cisgender women, transgender men, and intersex individuals assigned female at birth]) between September 27 and October 26, 2022. Participants who did not fall within the binary (transgender and intersex individuals) were included based on sex assigned at birth [31].

### Study procedures and sample collection

All potential participants were approached during their regularly scheduled clinical appointments on Mondays to Wednesdays, as this is when nearly all follow-up patients are attended to at this clinic. Inclusion criteria were adults (≥18 years) who attended the ART clinic during the study period and days assigned; exclusion criteria included those who were not living with HIV. As previously described [31], the minimum sample size was calculated based on the primary outcome of interest of expected genital chlamydia prevalence (not reported here) of 19% among AMaB and 10% among AFaB, assuming 3000 AMaB patients and 1200 AFAB patients attending the clinic bi-annually, and using a 95% confidence interval. Of all potential participants approached to attend, 76% of AMaB attendees and 82% of AFaB attendees gave written, informed consent in Spanish to participate. If individuals had difficulties reading or signing their name, a witness from outside the study team of their choosing was used; consent procedures for the signing with a fingerprint and witness signature were used. Data were obtained using a self-administered questionnaire in Spanish on a tablet computer using Kobo Toolbox software (Harvard Humanitarian Initiative, Cambridge, USA). The study personnel were available to help fill out the questionnaire if the participant could not.

### Measures

#### Adherence and retention in care measures

Suboptimal adherence (≥1 missed doses of ART in the past four weeks [>95% adherence]) and limited retention in HIV care (≥1 missed appointments at ART clinic in the past 12 months), measures based on current literature for the need to maintain near-perfect adherence [25, 27], and to maintain no missed visits within one year [32].

*Sociodemographic measures* included in the questionnaire included: gender (man, woman, another gender), age, ethnic group, employment, bimonthly salary, salary above or below the poverty line, type of housing, current cohabitation, difficulty paying rent in the last 12 months, moving with people due to difficulty paying rent in the previous 12 months, food security in the last 12 months, and balanced diet in the previous 12 months.

#### HIV diagnosis and management measurements

years since HIV diagnosis, years taking ART, time it takes to travel to the ART clinic.

*Sexual biography measures* gender identity (men, women, another gender), gender modality (cis-gender, transgender, or another modality), sexual identity (gay/lesbian, heterosexual, bisexual, another identity)

#### Behavioral measures

current cigarette use, current alcohol consumption, and other drug use currently (marijuana, crack, coke, heroin, hallucinogens, or a derivate of one of these).

*Mental health and interpersonal violence*: depressive symptoms with Patient Health Questionnaire (PHQ-9), lifetime emotional violence by an intimate partner (at some time, have had a partner that has insulted you, called you inappropriate names, or in some way belittled you?), lifetime physical violence by an intimate partner (at some time, have you had a partner that has hit you, scratched you?) Have you ever had a partner who has become upset or made you feel bad if you did not have sex with them?; lifetime forced sex by an intimate partner (have you at some time had a partner who has forced you to have sex when you did not want to?).

### Statistical methods

Questionnaire responses were uploaded into the Kobo Toolbox cloud, imported, and analyzed using Stata V18.0 (StataCorp, College Station, TX, USA). For ethical reasons, the questions in the questionnaire were voluntary and optional, and some individuals did not answer all questions. Therefore, data from individuals who did not answer specific questions were excluded from the specific analyses. Two individuals, one assigned male and one female at birth, were excluded from all analyses due to not answering the gender identity question. The χ^2^ test was used to evaluate the differences in sociodemographic, behavioral, mental health, and violence outcomes by gender identity. Sociodemographic, behavioral, mental health, and violence variables were then presented by outcome measures of low ART adherence and limited retention in HIV care. Kruskal Wallis Test was used to evaluate associative trends among variables. Logistic regression was used to calculate odds ratios (OR) and 95% confidence intervals (CI). Models were checked for collinearity using variance inflation factor. Variables associated with each outcome, at p<0.2, were included in the initial multivariable model adjusting for participant gender. The model first included sociodemographic (distal) variables, followed by behavioral (proximal) variables. The final model reported variables independently associated with the outcomes at p<0.05.

### Ethical procedures

This research was approved by the Gorgas Memorial Institutional Bioethics Board (628/CBI/ICGES/19). All participants were 18 years and older and gave written informed consent to participate.

## Results

### Sociodemographic factors

A total of 375 participants were included (209 who identified as men, 158 as women, and eight as another gender; **Table 1**).

**Table 1:** Demographic factors, by self-reported gender, among people who attend a large ART clinic in Panama City, Panama, 2022.

		All participants n/N (%)	Man n/N (%)	Woman n/N (%)	Another gender n/N (%)	p-value (difference between genders)
Age group		N = 375	N = 209	N = 158	N = 8	P<0.01
	18–30	122 (32.5)	73 (34.9)	45 (28.5)	4 (50.0)	
	31–40	117 (31.2)	66 (31.6)	50 (31.6)	1 (12.5)	
	>40	136 (36.3)	70 (33.5)	63 (39.9)	3 (37.5)	
Ethnic group		n = 373	N = 209	N = 156	N = 8	P<0.01
	Afro-descendent	72 (19.3)	37 (17.7)	35 (22.4)	0 (0.0)	
	White	67 (18.0)	44 (21.0)	23 (14.7)	0 (0.0)	
	Mestizo	180 (48.3)	97 (46.4)	80 (51.3)	3 (37.5)	
	Indigenous	52 (13.9)	30 (14.3)	18 (11.5)	4 (50.0)	
	Asian	2 (0.005)	1 (0.5)	0 (0.0)	1 (12.5)	
Years of living with HIV		N = 375	N = 209	N = 158	N = 8	P = 0.02
	<1 years	64 (17.1)	40 (19.1)	20 (12.7)	4 (50.0)	
	1–5 years	120 (32.0)	72 (34.5)	47 (29.8)	1 (12.5)	
	>5 Years	191 (50.9)	97 (46.4)	91 (57.6)	3 (37.5)	
The time it takes to travel to the ART clinic.		N = 374	N = 209	N = 1	N = 7	P = 0.01
	Less than 1 hour	149 (39.8)	92 (44.0)	56 (35.4)	1 (14.3)	
	1–2 hours	162 (43.3)	94 (44.5)	65 (41.1)	3 (42.9)	
	More than 2 hours	63 (16.8)	23 (11.0)	37 (23.4)	3 (42.9)	
Sexual identity		N = 364	N = 205	N = 151	N = 8	P<0.01
	Gay/lesbian	119 (32.7)	112 (54.6)	3 (2.0)	4 (50.0)	
	Heterosexual	181 (49.7)	47 (22.9)	133 (88.1)	1 (12.5)	
	Bisexual/another orientation	64 (17.6)	46 (22.4)	3 (37.5)	3 (37.5)	
Currently employed		N = 386	N = 203	N = 158	N = 7	P<0.01
	No	230 (62.5)	110 (54.2)	115 (72.8)	5 (71.4)	
	Yes	138 (37.5)	93 (45.8)	43 (27.2)	2 (28.6)	
Of those who work, salary every two weeks		N = 133	N = 89	N = 42	N = 2	P = 0.31
	USD$<500	87 (65.4)	53 (59.6)	32 (76.2)	2 (100.0)	
	USD$501–1000	34 (25.6)	26 (29.2)	8 (19.1)	0 (0.0)	
	USD$>1000	12 (9.0)	10 (11.2)	2 (4.5)	0 (0.0)	
Below poverty line		N = 362	N = 198	N = 157	N = 7	P<0.02
	No	123 (34.0)	83 (41.9)	39 (24.8)	1 (14.3)	
	Yes	239 (66.0)	115 (58.1)	118 (75.2)	6 (85.7)	
Where do you live		N = 372	N = 206	N = 158	N = 8	P = 0.69
	Apartment	86 (23.1)	52 (25.2)	32 (20.3)	2 (25.0)	
	House	253 (68.0)	137 (66.5)	110 (69.6)	6 (75.0)	
	Other	33 (8.9)	17 (8.3)	16 (10.1)	0 (0.0)	
In the last 12 months, did you have difficulty paying rent, mortgage, or utilities?		N = 371	N = 206	N = 157	N = 8	P = 0.572
	No	202 (54.5)	111 (53.9)	88 (56.1)	3 (37.5)	
	Yes	169 (45.6)	95 (46.1)	69 (44.0)	5 (62.5)	
In the last 12 months, participant moved in with friends or family because paying rent, mortgage, or utilities was difficult.		N = 370	N = 205	N = 157	N = 8	P<0.01
	No	305 (82.4)	161 (78.5)	140 (89.2)	4 (50.0)	
	Yes	65 (17.6)	44 (21.5)	17 (10.8)	4 (50.0)	
Was the food not enough in the last 12 months		N = 366	N = 203	N = 155	N = 8	P = 0.08
	Often	48 (13.1)	28 (13.8)	19 (12.3)	1 (12.5)	
	Sometimes	172 (47.0)	83 (40.9)	86 (55.5)	3 (37.5)	
	Never	146 (39.0)	92 (45.3)	50 (32.3)	4 (50.0)	
Current cigarettes use		N = 206	N = 206	N = 157	N = 8	P = 0.01
	No	329 (88.7)	175 (85.0)	148 (94.3)	6 (75.0)	
	Yes	42 (11.3)	31 (15.0)	9 (5.7)	2 (25.0)	
Alcohol consumption		N = 372	N = 206	N = 158	N = 8	P<0.01
	No	233 (62.6)	99 (48.1)	130 (82.3)	4 (50.0)	
		N = 139	N = 107	N = 28	N = 4	P<0.02
	Yes- once a day or more	3 (2.2)	1 (0.9)	1 (3.6)	1 (25.0)	
	Yes- multiple times a week	3 (2.2)	3 (2.8)	0 (0.0)	0 (0.0)	
	Once a week/once a month, or very little	133 (95.7)	103 (96.3)	27 (96.4)	3 (75.0)	
Use of another drug (Marihuana, crack, coke, heroin, hallucinogens, or a derivate of these)						
	No					
	Yes	N = 373	N = 207	N = 158	N = 8	P = 0.27
Depressive symptoms						
	0–9 points (minimal to mild)	37 (9.9)	25 (12.1)	11 (7.0)	1 (12.5)	
	10–19 points Moderate/moderately severe	162 (43.4)	95 (45.9)	64 (40.5)	3 (37.5)	
	20 and above points severe	174 (46.6)	89 (42.0)	83 (52.5)	4 (50.0)	
Lifetime Emotional violence		N = 374	N = 209	N = 157	N = 8	P = 0.02
	No	254 (67.9)	153 (73.2)	98 (62.4)	3 (37.5)	
	Yes	120 (32.1)	56 (26.8)	59 (37.6)	5 (62.5)	
Lifetime Physical violence		N = 375	N = 209	N = 158	N = 8	P = 0.003
	No	255 (68.0)	156 (74.6)	96 (60.8)	3 (37.5)	
	Yes	120 (32.0)	53 (25.4)	62 (39.2)	5 (62.5)	
Partner has gotten upset participant didn’t want to have sex with them.		N = 375	N = 209	N = 158	N = 8	
	No	251 (66.9)	145 (69.4)	100 (63.3)	6 (75.0)	P = 0.42
	Yes	124 (33.1)	64 (30.6)	58 (36.7)	2 (25.0)	
Lifetime forced sex		N = 375	N = 209	N = 158	N = 8	P = 0.04
	No	308 (82.1)	180 (86.1)	123 (77.9)	5 (62.5)	
	Yes	67 (17.9)	29 (13.9)	35 (22.1)	3 (37.5)	

### Adherence and retention in care

In total, 37.3% (125/335) participants reported suboptimal adherence, and this was reported by 28.6% (n = 53/185) of men, 49.0% (n = 71/145) women, and 20.0% (n = 1/5) individuals of another gender; (p<0.01). In total, 18.6% (n = 69/371) of the participants reported limited retention in care, and this was reported in 13.6% (28/206) of men, 24.2% (38/157) of women, and 37.5% (3/8) of people of another gender (p = 0.01). In all, there were 8.4% (28/335) of participants who reported both suboptimal adherence and limited retention in HIV care.

Of those (N = 40) who did not respond to the adherence question, 47.4% were 18–39 years, 23.7% 31–40 years, 29.0% >40 years (difference in age with those who responded, p = 0.12), 60% (n = 24) were men, 32.5% (n = 13) were women, and 7.5% (n = 3) were of another gender (difference with those who responded, p = 0.01); 45% (n = 18) had their diagnosis within one year before sampling, 22.5% (n = 9) within 1–5 years, 32.5% (n = 13) more than five years (difference with those who responded, p<0.01).

### Factors associated with suboptimal adherence and limited retention in care

In unadjusted analyses (**Table 2**), suboptimal adherence was associated with gay/lesbian sexual identity 44.3% vs. heterosexual identity 26.2%, OR = 2.24, 95%CI:1.32–3.81, emotional violence by an intimate partner 48.2% vs. no emotional violence 32.2%, OR = 1.96, 95%CI: 1.15–2.90, lifetime physical violence by an intimate partner 46.9% vs. no violence 32.6%, OR = 1.82, 95%CI: 1.15–2.90, and if their partner got upset if they did not have sex with them 46.1% vs. not getting upset 32.9% OR = 1.74, 95%CI: 1.10–2.77). In multivariable analyses, suboptimal adherence was associated with gender: women 49.0% vs. men 28.6%, AOR = 1.86, 95%CI:0.97–3.57, and severe depressive symptoms (46.2% vs 28.1% who had suboptimal adherence with minimal to mild rating, AOR = 2.19, 95%CI:0.96–5.04).

**Table 2:** Factors related to suboptimal adherence and limited retention in care among people attending a large ART clinic in Panama City, Panama, 2022.

		Suboptimal adherence (95%)	P-value	OR suboptimal adherence	AOR suboptimal adherence	Limited retention	P-value	OR Limited retention	AOR Limited retention
Gender identity			0.001				0.01		
	Men	53/185 (28.6)		1		28/206 (13.6)		1	1
	Women	71/145 (49.0)		**2.39 (1.51–3.77)**	**1.86 (0.97–3.57)**	38/157 (24.2)		**2.03 (1.18–3.49)**	0.73 (0.16–3.29)
	Another gender	1/5 (20.0)		0.62 (0.07–5.70)	0.54 (0.06–5.28)	3/8 (37.5)		3.81 (0.86–16.85)	-
Gender modality			0.46				0.124		
	Cis-gender	123/327 (37.6)		1		65/360 (18.1)		1	
	Transgender or another gender	2/8 (25.0)		0.55 (0.11–2.78)		4/11 (36.4)		2.59 (0.74–9.12)	
Age group (years)			0.60						
	18–30	35/104 (33.6)		1		21/121 (17.4)	0.88	1	
	31–40	43/107 (40.2)		1.32 (0.76–2.09)		23/116 (19.8)		1.78 (0.61–2.27)	
	>40	48/126 (38.1)		1.21 (0.70–2.09)		26/136 (19.1)		1.12 (0.59–2.16)	
Ethnic group			0.70				0.70		
	Mixed	30/67 (44.8)		1		30/67 (44.8)		1	
	White	22/63 (34.9)		0.66 (0.33–1.34)		22/63 (34.9)		0.57 (0.22–1.47)	
	Afro-descendent	59/162 (36.4)		0.71 (0.39–1.26)		59/162 (36.4)		1.00 (0.50–1.99)	
	Indigenous	14/42 (33.3)		0.62 (0.28–1.37)		14/42 (33.3)		1.30 (0.54–3.10)	
	Asian	½ (50.0)		0.62 (0.07–20.55)		½ (50.0)		4.21 (0.25–71.58)	
Years of living with HIV			0.65				0.01		
	<1 years	16/46 (34.8)		1		4/61 (6.6)		1	1
	1–5 years	39/112 (34.8)		1.00 (0.49–2.06)		21/120 (17.5)		**3.02 (0.99–9.24)**	0.97 (0.32–2.99)
	>5 Years	71/179 (39.7)		1.23 (0.63–2.42)		45/192 (23.4)		**4.36 (1.50–12.68)**	-
Sexual identity			0.10				<0.01		
	Heterosexual	28/107 (26.2)		1	1	10/119 (8.4)		1	1
	Gay/lesbian	74/167 (44.3)		**2.24 (1.32–3.81)**	0.73 (0.35–1.51)	32/179 (17.9)		**2.98 (1.12–5.03)**	0.21 (0.04–1.11)
	Bisexual/another orientation	21/55 (38.2)		1.74 (0.87–3.49)	0.86 (0.40–1.82)	25/64 (39.1)		**6.99 (3.10–15.86)**	1.41 (0.28–6.99)
Currently employed			0.75				0.879		
	No	78/204 (38.2)		1		42/229 (18.3)		1	
	Yes	46/126 (36.5)		0.92 (0.58–1.47)		26/137 (19.0)		1.04 (0.61–1.79)	
Of those who work, salary every two weeks			0.06				0.06		
	USD$<500	36/80 (45.0)		0.93 (0.09–10.04)		36/80 (45.0)		0.64 (0.11–3.61)	1.63 (0.22–11.97)
	USD$501–1000	8/31 (25.8)		1		8/31 (25.8)		1	1
	USD$>1000	2/11 (18.2)		0.76 (0.18–3.23)		2/11 (18.2)		**2.35 (0.94–5.89)**	1.30 (0.35–4.81)
Below poverty line			0.61				0.50		
	No	41/114 (36.0)		1		20/122 (16.4)		1	
	Yes	82/211 (38.9)		1.13 (0.38–1.81)		46/238 (19.3)		1.22 (0.69–2.18)	
Where participant lives			0.73				0.61		
	Apartment	32/76 (42.1)		1		13/85 (15.3)		1	
	House	81/231 (35.1)		0.74 (0.44–1.26)		50/252 (19.8)		1.37 (0.70–2.67)	
	Other	12/27 (44.4)		1.1 (0.45–2.67)		7/33 (21.2)		1.49 (0.54–4.15)	
In the last 12 months, participant has had difficulty paying rent, mortgage, or utilities.			0.62				0.06		
	No	66/183 (36.1)		1		30/201 (14.9)		1	1
	Yes	58/150 (38.7)		1.12 (0.72–1.74)		38/168 (22.6)		**1.67 (0.98–2.83)**	0.68 (0.23–2.03)
In the last 12 months, participant had to move in with friends or family because paying rent, mortgage, or utilities was difficult			0.32				0.16		
	No	99/274 (36.1)		1		52/303 (17.2)		1	
	Yes	25/58 (43.1)		1.34 (0.75–2.38)		16/65 (24.6)		1.57 (0.83–2.98)	
Was the food not enough in the last 12 months			0.35						
	Often	18/39 (46.1)		1		11/48 (22.9)	0.55	1	
	Sometimes	57/153 (37.2)		0.69 (0.34–1.41)		28/172 (16.3)		0.65 (0.30–1.43)	
	Never	46/137 (33.6)		0.60 (0.30–1.21)		27/144 (18.8)		0.78 (0.35–1.71)	
In the last year, participant could not maintain a balanced diet.			0.06						
	Often	31/63 (49.2)		1		14/77 (18.2)	0.99	1	
	Sometimes	54/158 (34.2)		0.54 (0.30–0.97)		32/172 (18.6)		1.03 (0.51–2.06)	
	Never	35/108 (32.4)		0.49 (0.26–0.94)		21/116 (18.1)		1.00 (0.47–2.10)	
Current cigarette smoking			0.005				0.24		
	No	102/295 (34.6)		1		59/330 (17.9)		1	
	Yes	22/38 (57.9)		2.60 (1.31–5.17)		10/39 (25.6)		1.58 (0.73–3.42)	
Current alcohol consumption			0.26						
	No	82/206 (39.8)		1					
	Yes, at any frequency	43/128 (33.6)		0.76 (0.48–1.21)					
Current use of another drug (Marihuana, crack, coke, heroin, hallucinogens, or a derivate of these)			0.29				0.25		
	No	117/318 (36.8)		1		64/353 (18.1)		1	
	Yes	8/16 (50.0)		1.72 (0.63–4.70)		5/17 (29.4)		1.88 (0.64–5.53)	
PHQ-9 score			0.006				0.28		
	0–9 points (minimal to mild)	9/32 (28.1)		1	1	4/37 (10.8)		1	
	10–19 points Moderate/moderately severe	43/145 (29.7)		0.88 (0.36–2.14)	1.08 (0.46–2.52)	28/161 (17.4)		1.74 (0.57–5.30)	
	20 and above points Severe	73/158 (46.2)		1.46 (0.59–3.57)	**2.19 (0.96–5.04)**	37/173 (21.4)		2.24 (0.75–6.74)	
Emotional violence				0.004			0.18		
	No	73/227 (32.2)		1	1	42/252 (16.7)		1	
	Yes	53/110 (48.2)		**1.96 (1.23–3.13)**	1.50 (0.80–2.81)	27/120 (22.5)		1.45 (0.84–2.49)	
Physical violence			0.10				0.32		
	No	73/224 (32.6)		1	1	44/253 (17.4)		1	
	Yes	53/113 (46.9)		**1.82 (1.15–2.90)**	1.16 (0.63–2.14)	26/120 (21.7)		1.31 (0.76–2.26)	
Partner has gotten upset the participant didn’t want to have sex with them.			0.02				0.66		
	No	73/222 (32.9)		1	1	45/248 (18.2)		1	
	Yes	53/115 (46.1)		**1.74 (1.10–2.77)**	1.15 (0.66–2.02)	25/125 (20.0)		1.23 (0.65–1.94)	
Forced sex			0.22				0.88		
	No	99/276 (35.9)		1		57/306 (18.6)		1	
	Yes	27/61 (44.3)		1.42 (0.81–2.49)		13/67 (19.4)		1.05 (0.53–2.06)	

In unadjusted analyses, limited retention in care was associated with gender: women (24.2% compared men 13.6% OR = 2.03, 95%CI:1.18–3.49), having lived longer after HIV diagnosis: 1–5 years 17.5% vs. less than one year (6.6%), OR = 3.02, 95%CI: 0.99–9.24) and more than five years 23.4%, OR = 4.36, 95%CI: 1.50–12.68), having difficulty paying rent, mortgage or utilities in the past 12 months (22.6% vs. 14.9% who did not have difficulty paying rent or mortgage, OR = 1.67, 95%CI = 0.98–2.83). No variables were associated with limited retention in the multivariable model.

## Discussion

Panama is lagging in reaching UNAIDS’ 95-95-95 goals; in 2022, only 71% of individuals who were on ART maintained viral suppression, and of all estimated people to be living with HIV [20]. The present results suggest that this may be in part due to suboptimal adherence and limited retention in HIV care, which are associated with gaps related to gender and psychosocial determinants of health. This analysis is the first study to examine sociodemographic and psychosocial factors related to suboptimal adherence and limited retention in HIV care among people attending a large urban ART clinic in Panama City, Panama. Despite the importance of near-perfect ART, even with newer TLD regimens, in our study, over a third of participants reported ART adherence of <95%, and over a quarter of participants had limited retention in HIV care. Suboptimal adherence and limited retention were more common among women, gay/lesbian individuals, individuals with unstable housing, those with elevated depressive symptoms, and survivors of emotional and physical intimate partner violence. Our findings indicate the importance of the social determinants of health on ART adherence and retention in HIV care.

UNAIDS has recognized gender inequalities as a barrier to reaching 95-95-95 goals. Our study revealed that gender identity affects ART adherence and retention in HIV care and contributes to a growing literature from other geographic locations [33–35]. Since 2010 in Panama, there has been a 36.8% increase in HIV-related mortality among women and a 32.9% decrease in HIV-related mortality among men [36]. Traditionally, women often bear a disproportionate burden of caregiving responsibilities in the home. Similar to healthcare access in general, women who seek HIV care may have difficulties reaching appointments due to several social and structural factors [37, 38], including household responsibilities and limited financial and socioeconomic agency [39, 40]. Due to these disparities, healthcare must be flexible to meet these needs by rescheduling appointments. While men generally had higher adherence than women, the literature suggests that Latino men face barriers to care, such as culturally specific masculine norms (*machismo*) and stigma [16, 41].

We found that ART non-adherence was associated with depression symptoms; this finding has been encountered worldwide, especially among women [35, 42–44]. Longitudinal studies suggest this relationship may be bi-directional [45]. Findings from the current study hint at elevated barriers to ART adherence and retention in HIV care among transgender and other gender-diverse populations [46, 47], including higher rates of mental health concerns [48] and a greater number of experiences of intimate or community violence [49]. Future work in Panama should seek to over-sample gender-diverse populations and examine unique factors transgender populations may face.

We found high prevalence of lifetime intimate partner violence, especially among women and individuals who identify outside of the gender binary. Previous studies across Panama also found high prevalence among boy and girl adolescents; together with the current study, these data indicate a widespread problem across the country [50, 51]. In the current study, IPV was further associated with suboptimal adherence and, to some extent, limited retention in HIV care, which has also been observed in sub-Saharan Africa [52–54]. Our study showed that, in addition to those who identified as women, people who identified outside the gender binary were significantly affected by IPV. Though data is limited for non-binary populations, a meta-analysis found no statistical difference between non-binary and binary transgender individuals (e.g., transgender women), suggesting that non-binary individuals also have elevated IPV similar to their binary transgender counterparts [49].

Our findings also found evidence of an association between minority sexual identity and limited adherence to and retention in care. For example, we found that those who reported gay/lesbian identity were more likely to have suboptimal antiretroviral adherence, and those who reported gay/lesbian or bisexual identities had limited retention in care. Sexual minorities may face perceived and internalized stigma owing to sexual identity and HIV status [55, 56]. These stigmas may negatively affect provider-patient interactions and limit ART adherence and retention in care. Patients often face higher levels of stigma and discrimination, which can hinder their engagement with healthcare services, possibly due to medical mistrust [56].

### Program and policy implications

The findings of this study could have program and policy implications for managing HIV care services in urban areas in Panama. First, healthcare programs should be adapted to incorporate gender-sensitive interventions, as well as interventions to reduce multiple and intersecting forms of violence for PLHIV [9]. Second, efforts should also focus on providing mental health services in all ART clinics in Panama [57, 58]. Depression screening is not mandatory in ART clinics in Panama, and limited ART clinics have mental health professionals. Interventions across Panamanian HIV clinics could include individual and group psychosocial interventions with cognitive behavioral therapy/mindfulness-based cognitive behavioral therapy [59, 60]. Community-based differentiated ART service models, such as out-of-facility care and ART dispensing, could increase ART adherence, especially for those with increased social and structural disparities [61, 62]. Additional provisions that could reduce barriers to care should be considered, such as telehealth-based therapy [59, 63]. Stigma may be addressed through group cognitive behavior therapy [64] or training culturally competent healthcare professionals. Lastly, employing new technologies, such as longer-acting injectable regimens, could revolutionize HIV care in the country by limiting the burden on patients due to daily pill requirements [65].

### Limitations

This cross-sectional study’s strengths include the use of self-administered questionnaires and decent acceptance of participation among study participants [31]. Self-reporting for questions that are difficult to discuss for social reasons is advantageous for decreasing social desirability bias; furthermore, self-reported adherence and retention may be prone to recall and reporting bias. Although all data collection occurred during 2022, individuals assigned male and female at birth were included at different periods of the year due to biological testing funding restrictions (not presented here). However, there were no programmatic changes made during 2022 in Panama to increase or change adherence and retention in HIV care, which could cause significant differences in adherence and retention indicators between genders. Additionally, due to the voluntary nature of this study and for ethical reasons, participants were not obligated to respond to all questions. Therefore, some variables, and notably the adherence outcome variable, have missing values if participants did not answer; importantly the variables of gender and number of years since HIV diagnosis were significantly different between those who responded and those who did not. Lastly, the study’s cross-sectional design limits the results to associations yet does not describe causality. Despite these shortcomings, this study had a decent sample size to describe some psychosocial factors related to suboptimal ART adherence and limited retention in care.

## Conclusions

This study described the prevalence of suboptimal ART adherence, limited retention in care, and some psychosocial determinants that may impact viral suppression and eventually the survival of PLHIV attending a large ART clinic in Panama City. These determinants included the gender of the individual, sexual identity, depression, and IPV. The findings of this study could initiate a roadmap for developing effective interventions, including mental health support and IPV prevention and intervention services. As Panama strives to achieve UNAIDS’ 95-95-95 targets, these findings provide a crucial foundation for identifying gaps in adherence and retention in care. This understanding is essential for promoting equitable healthcare and guiding the development of evidence-based policies and programs for PLHIV.
